# 3-dimensional electron microscopic imaging of the zebrafish olfactory bulb and dense reconstruction of neurons

**DOI:** 10.1038/sdata.2016.100

**Published:** 2016-11-08

**Authors:** Adrian A. Wanner, Christel Genoud, Rainer W. Friedrich

**Affiliations:** 1 Friedrich Miescher Institute for Biomedical Research, Maulbeerstrasse 66, 4058 Basel, Switzerland; 2 University of Basel, 4003 Basel, Switzerland

**Keywords:** Scanning electron microscopy, Neural circuits, Olfactory bulb

## Abstract

Large-scale reconstructions of neuronal populations are critical for structural analyses of neuronal cell types and circuits. Dense reconstructions of neurons from image data require ultrastructural resolution throughout large volumes, which can be achieved by automated volumetric electron microscopy (EM) techniques. We used serial block face scanning EM (SBEM) and conductive sample embedding to acquire an image stack from an olfactory bulb (OB) of a zebrafish larva at a voxel resolution of 9.25×9.25×25 nm^3^. Skeletons of 1,022 neurons, 98% of all neurons in the OB, were reconstructed by manual tracing and efficient error correction procedures. An ergonomic software package, PyKNOSSOS, was created in Python for data browsing, neuron tracing, synapse annotation, and visualization. The reconstructions allow for detailed analyses of morphology, projections and subcellular features of different neuron types. The high density of reconstructions enables geometrical and topological analyses of the OB circuitry. Image data can be accessed and viewed through the neurodata web services (http://www.neurodata.io). Raw data and reconstructions can be visualized in PyKNOSSOS.

## Background & Summary

The dense anatomical reconstruction of neuronal populations is a major goal in neuroscience because it provides detailed structural information about neuronal cell types, circuits and wiring diagrams. Such information is important to understand the diversity of neuron types and the organization of neuronal networks^1–17^. Given that neuronal processes can be long, geometrically complex, and <100 nm in diameter, imaging methods for dense neuronal reconstructions need to provide ultrastructural resolution throughout large volumes. These goals can be achieved by volumetric electron microscopy (EM) approaches based on serial sectioning^1,13,18–20^. We used serial block face EM (SBEM), an approach that is based on the operation of an automated ultramicrotome inside the vacuum chamber of a scanning electron microscope^1,6,18^. Stacks of images are acquired by repeated removal of ultrathin sections (typically <30 nm) from a sample block and imaging of the block face. Brain tissue is impregnated with heavy metals to generate contrast of lipid membranes and other structures including synaptic densities^21,22^. To alleviate problems caused by charging of the sample^18,23–25^ we introduced conductive silver particles into the resin surrounding the sample. This approach increased the signal to noise ratio because images could be acquired in high vacuum^26^.

Tracing of neuronal processes in 3D image data is necessary to obtain geometrical representations of neurons. This process is challenging and requires substantial human input because fully automated image segmentation methods do not yet achieve sufficiently high accuracy. We traced neurons manually by placing connected nodes onto cross-sections of neurites in the image data. Tracing was performed in KNOSSOS^27^ or PyKNOSSOS^26^, two software tools designed specifically for high-throughput 3D image annotation and neuron reconstruction. The bulk of skeleton tracing tasks was outsourced to a company (www.ariadne-service.ch). Each neuron was initially reconstructed by three different individuals (‘tracers’). Skeletons were then compared and corrected iteratively by ‘COnvergence by Redundancy and Experts’ (CORE), a procedure that involves local re-tracing around mismatch points and focused input from a scientific expert. This workflow is efficient and achieves high accuracy^26^.

We analyzed the organization of neurons in the olfactory bulb (OB), which receives sensory input through an array of discrete neuropil units, the glomeruli. In adult vertebrates, each glomerulus receives input from olfactory sensory neurons expressing the same odorant receptor^28,29^. Principal neurons of the OB, the mitral/tufted cells, receive input from olfactory sensory neurons and provide output to multiple target areas of the OB. Within the OB, mitral/tufted cells interact via different types of GABAergic interneurons (INs)^30^. In the adult OB, the most prominent IN types are periglomerular and short axon cells in the upper layers and granule cells in the deep layers. INs are critical for neuronal computations such as the equalization and decorrelation of odor-evoked activity patterns^30–33^ but the topological organization of IN networks has been poorly understood. Moreover, most INs arise late in development^34,35^ but it has been unclear how IN networks change as the OB matures.

**Figure 1:**
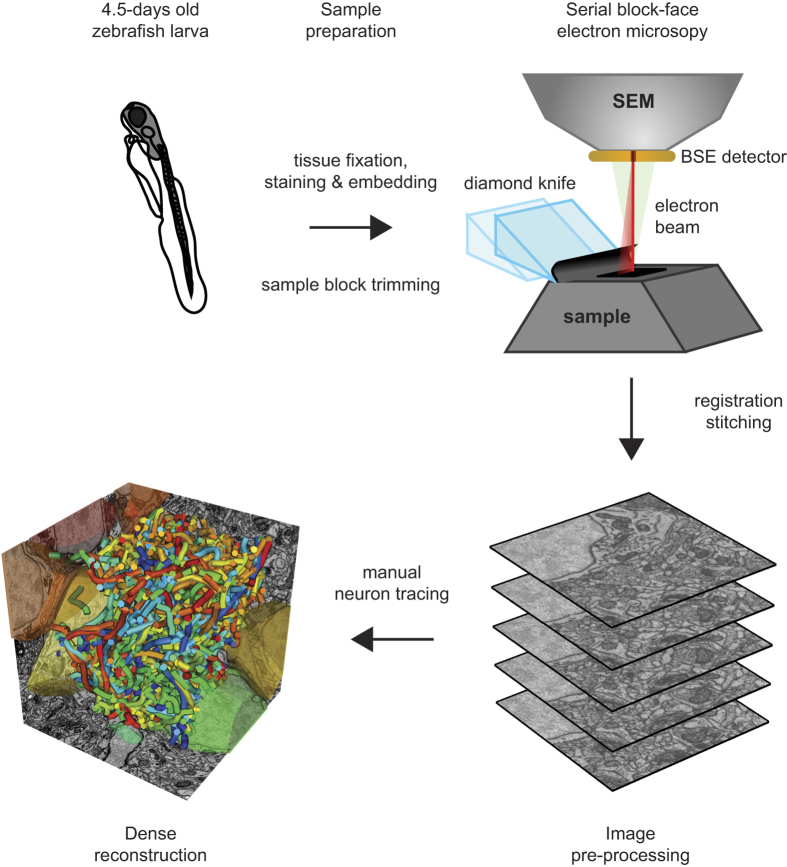
Schematic illustration of workflow for sample preparation, SBEM imaging and neuron reconstruction.

**Figure 2:**
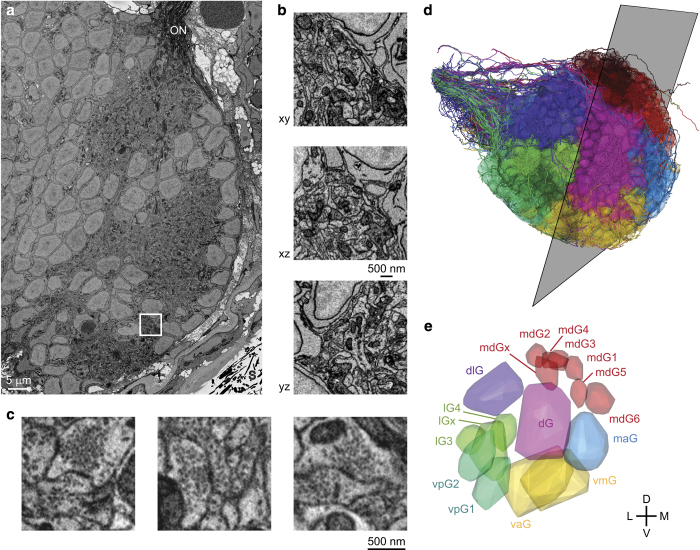
SBEM. (**a**) EM image showing cross-section through the OB. ON, olfactory nerve; S, silver particles. (**b**) Reslices through a subvolume. (**c**) Examples of synapses. (**d**) Orientation of image plane in (**a**) is illustrated relative to the reconstruction of all 1,022 neurons in the OB (Dataset 2). Colors depict the group of the parent glomerulus (longest neurite length). (**e**) 3D outlines of the 17 glomeruli in the OB (Dataset 3). Colors depict different groups of glomeruli as defined by Braubach and colleagues^47,48^. 3D rendering in (**d**,**e**) was done in PyKNOSSOS. Parts of this figure have been modified from illustrations in a previous publication^26^.

To address these and additional questions we acquired a stack of SBEM images of the OB in a zebrafish larva and reconstructed approximately 98% of all OB neurons (*n*=1,022; Figs 1 and 2). The results identified novel rare cell types and indicate that granule cells develop later than other IN types. Inter-glomerular projections were mediated primarily by INs and formed a specific pattern that is governed by glomerular identity, as defined by the associated odorant receptors. The datasets allow for multiple additional analyses including further morphological analyses of individual neurons, geometrical analyses of glomeruli and neuronal populations, ultrastructural analyses of synapses in different cell types, other ultrastructural analyses, and analyses of neuronal subpopulations associated with specific glomeruli. Moreover, the high density and accuracy of skeleton reconstructions may be useful for the development and validation of procedures for automated segmentation of volumetric EM image data^7,8,36–39^. Data are accessible through the neurodata web services of the open connectome project (http://neurodata.io)^40^.

## Methods

Most methods have been described in detail in a previous publication^26^.

### Electron microscopy

#### Dissection

All animal procedures were approved by the Veterinary Department of the Canton Basel-Stadt (Switzerland). Fish were housed and bred in an animal facility under standard conditions. The sample was prepared from a zebrafish larva 4.5 days post fertilization (dpf) that expressed the genetically encoded calcium indicator GCaMP5 under the control of the panneuronal elavl3 promoter^41^. The sex is not yet determined at this developmental stage. Prior to sample preparation, the larva was paralyzed with Mivacron, mounted in low-melting agarose (Sigma Aldrich A9414), and immersed in standard E3 zebrafish medium^42^ (5 mM NaCl, 0.17 mM KCl, 0.33 mM CaCl_2_, 0.33 mM MgSO_4_, pH 7.4). Responses of olfactory bulb neurons to odor stimulation were then measured by multiphoton microscopy as described^43^ over a period of approximately 4 h. Subsequently, the larva was anesthetized in tricaine methanesulfonate (MS-222) and a small craniotomy was made above the contralateral olfactory bulb using a glass pipette to facilitate the penetration of reagents into the brain. Immediately afterwards the larva was transferred into EM fixative (Table 1).

#### Fixation and staining

The sample was fixed and stained *en bloc* using an established protocol with minor modifications^21,22^. The procedure includes successive exposures of the sample to reduced OsO_4_, thiocarbohydrazide (TCH), OsO_4_, uranyl acetate and lead aspartate. Reagents and solutions are described in Table 1. Tissue was initially fixed in EM fixative for 1 h at room temperature and 1 h on ice. After five washes (always 3 min each) with ice-cold cacodylate buffer, samples were postfixed in RedOs solution for 1 h on ice, washed 5 times in bidistilled H_2_0, and incubated in fresh TCH solution for 20 min at room temperature. In a second postfixation step, samples were incubated in Os solution for 30 min at room temperature and washed five times in bidistilled H_2_0. The sample was then incubated in UA solution and left in a refrigerator (4 °C) overnight, washed five times in bidistilled H_2_0, incubated at 60 °C for 30 min in bidistilled H_2_0, incubated in Walton’s lead aspartate for 20 min at 60 °C, and washed five times in bidistilled H_2_0 at room temperature. The sample was then dehydrated in an ethanol series (20%, 50%, 70%, 90%, 100%, 100%; 5 min each), incubated in 50% ethanol and 50% Epon resin for 30 min, and incubated in 100% Epon resin for 1 h at room temperature. Epon resin was exchanged and samples were again incubated at room temperature for 4–12 h (overnight).

#### Embedding and mounting of the sample

After fixation and staining the sample was embedded in a silver-filled epoxy to minimize charging of the sample block during exposure to the electron beam. The silver-filled epoxy was made from a commercially available 2-component glue that contains elongated silver particles with a size up to 45 μm (EE129-4; Epo-Tek). Deviating slightly from the instructions for normal use, the two components A and B were mixed in a ratio of 1.25:1. This ratio yielded conductive sample blocks with mechanical properties that allowed for reliable thin sectioning (25 nm). The sample was transferred to the conductive medium and moved gently to ensure that silver particles contact the surface of the tissue. The epoxy was then cured by incubation at 60 °C for 48 h. The resistance across the sample block was measured with an Ohm-meter and found to be <1 Ohm. The sample block was glued on an aluminum stub for SBEM (Gatan) using cyanoacrylate glue and trimmed to a pyramid with a block face area of approximately 300 μm×200 μm.

#### Electron microscopy

Images were acquired using a scanning electron microscope (QuantaFEG 200; FEI) equipped with an automated ultramicrotome inside the vacuum chamber (3View; Gatan). The ultramicrotome cut successive sections at a thickness of 25 nm. After each section, the sample block-face was scanned. The microscope, the stage and the ultramicrotome were controlled using DigitalMicrograph software (Gatan). Images were generated by detection of backscattered electrons with a silicon diode detector (Opto Diode Corp., USA). The signal was preamplified and further amplified by standard components of the 3View system (Gatan) before digitizing at 16 bit. The sample was scanned in high vacuum with a landing energy of 2 kV. Due to conductive embedding, artifacts arising from sample charging were negligible^26^. Pixel size was 9.25×9.25 nm^2^, the electron dose was 17.5 *e*^−^nm^−2^, and the pixel acquisition rate was 200 kHz. To cover the entire cross-section of the OB, multiple images (tiles) were acquired with a size up to 4,096×4,096 pixels and an overlap of 5–8%. Choosing a relatively small tile size allowed us to effectively avoid image distortions. The size and arrangement of tiles was adjusted to changes in the cross-section of the OB during acquisition of the stack. 4,746 successive sections were acquired over a period of 35 days. One section was lost due to technical problems (between slices 1301 and 1302). The focus was readjusted on average every 14.5 h. The final stack was cropped to a size of 72.2×107.8×118.6 μm^3^.

#### Image processing

Because distortions of raw images were minimized by conductive embedding and small tile size, registration of images in the stack could be achieved by simple translational alignment procedures. Pre-processing, registration and stitching of images was performed using custom software tools written in MATLAB that allowed for parallel batch processing of large datasets. For image registration, translational offsets between neighboring image tiles were calculated using a custom optimized cross-correlation procedure in MATLAB. Image columns were standardized by subtracting the mean and dividing by the standard deviation of the pixel intensities. The same standardization was subsequently applied to the rows. Translational offsets between neighboring images were calculated by determining the maximum 2D cross-correlation of the standardized images in the Fourier domain. The second inverse Fourier transform was restricted to the central region of the 2D cross-correlogram reflecting a maximal expected offset between overlapping image parts of 256 pixels. Using this procedure the offsets between two 1024×1024 pixel images with translational offsets of dX=69 and dY=66 pixels could be calculated in <0.36 s on a laptop computer (Intel Core i7-3520M CPU @ 2.90 GHz×4, 7.5 GB RAM, Ubuntu 12.10). Offsets were used to optimize the tile positions in a global total least square displacement sense. Image contrast was normalized by fitting a Gaussian distribution to the pixel intensity histogram and thresholding at 1.5–3 standard deviations around the peak of the Gaussian distribution to convert the images to 8 bit. Stacks were then divided into cubes of 128×128×128 voxels for dynamic data loading in KNOSSOS or PyKNOSSOS.

### Software: PyKNOSSOS

#### PyKNOSSOS: general description

**Figure 3:**
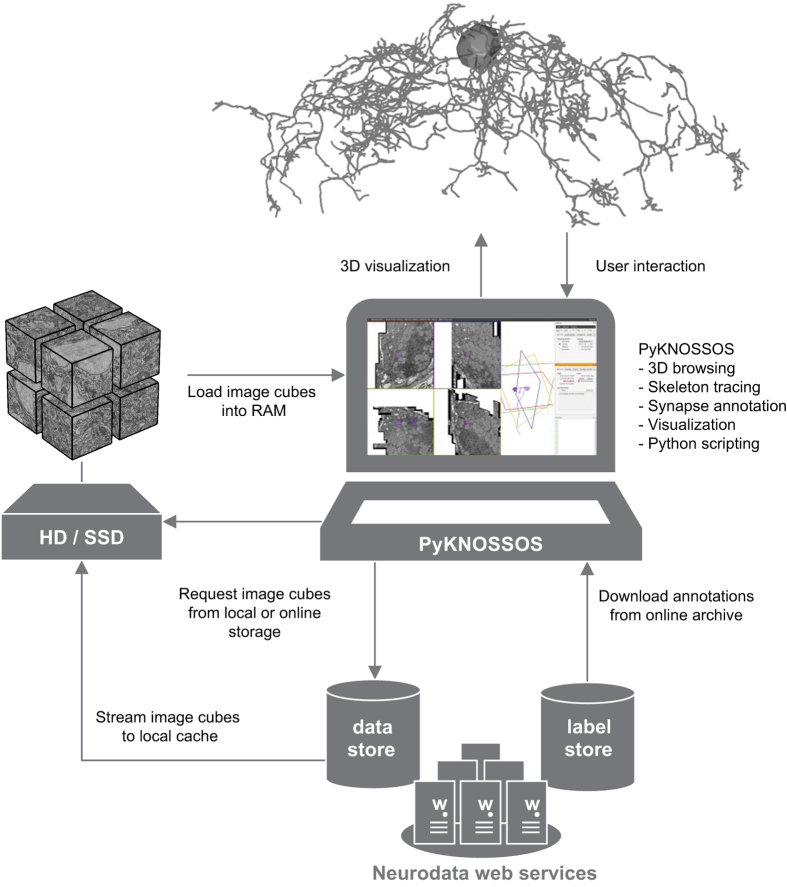
Access of data from web server using PyKNOSSOS. PyKNOSSOS running on a local machine streams cubed image data from a web server (data store; NeuroData) and stores cubes on disk. Cubes are generated by NeuroData and dynamically loaded into PyKNOSSOS. Annotations (e.g., skeleton reconstructions) are downloaded from a repository (label store) and may be modified locally.

**Figure 4:**
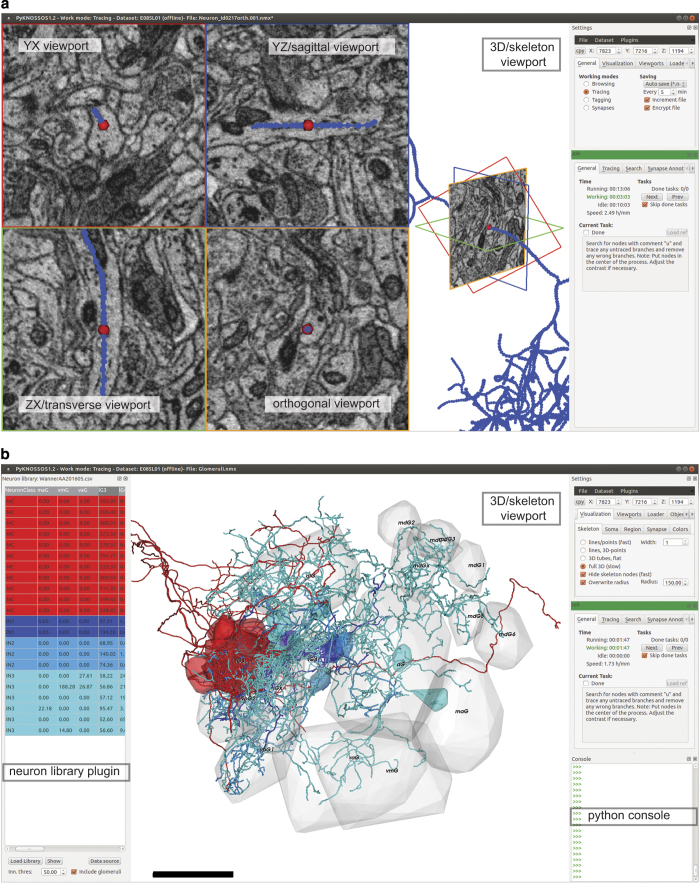
PyKNOSSOS. (**a**) Screenshot of PyKNOSSOS in tracing mode. (**b**) Screenshot of PyKNOSSOS using the NeuronLibrary plugin for visualization of specific neurons. The table on the left shows part of Dataset 4 (neurite length of each neuron in each glomerulus). Specific neurons were extracted by filtering, color-coded, and visualized together with 3D outlines of glomeruli (Dataset 3).

We developed PyKNOSSOS, a software package for ergonomic manual skeleton tracing, visualization and annotation of neurons in 3D image datasets (Figs 3 and 4). Other publicly available applications for similar purposes include KNOSSOS (www.knossostool.org)^27^, CATMAID (http://catmaid.org/)^44^ and ilastik (www.ilastik.org)^45^. PyKNOSSOS was inspired by KNOSSOS but is fully implemented in Python, which enables rapid and efficient code development, modification and extension. Efficient real-time 3D visualization of large numbers of reconstructed neurons together with raw image data is achieved using the VTK-Python bindings in combination with custom image data loading procedures written in C++. The user interface of PyKNOSSOS has been designed by close interactions between programmers and users to integrate user feedback into the application as directly as possible. PyKNOSSOS is written in Python (version 2.7) and runs on various operating systems including Linux and Windows 7. The main graphical user interface is based on the PyQT4 library. PyKNOSSOS can be run as stand-alone executable or directly from script using a Python interpreter. All source code and detailed installation instructions can be found on https://github.com/adwanner/PyKNOSSOS.

#### PyKNOSSOS: multi-scale dynamic data loading

Similar to KNOSSOS, PyKNOSSOS accesses a cubed version of the dataset in which the imaging data is divided into 8-bit cubes (Fig. 3). The default cube size is 128 pixel edge length but arbitrary cube dimensions can be processed. While navigating through the dataset, a custom C++ routine dynamically loads the image data in a pre-defined neighborhood around the current focal point into RAM. If multiple versions of the dataset at different zoom levels exist, PyKNOSSOS supports instantaneous seamless browsing and multi-scale zooming. At a given zoom level l_i_, a cubed neighborhood of typically 320–576 pixels around the current focal point is loaded into memory for all stored consecutive zoom levels l_i-1_, l_i_ and l_i+1_. This permits navigation through large datasets (>4 TB) with minimal RAM requirements (<4.5 GB). The data can be loaded from local hard disk storage, via a hybrid streaming pipeline from HTTP accessible servers, or via the JPEG stack service of the data API of neurodata (http://www.neurodata.io; Fig. 3).

#### PyKNOSSOS: built-in Python console and plugin interface

In development mode, PyKNOSSOS allows direct interaction and access to its core variables and functions via a built-in Python console. In addition, PyKNOSSOS features an easy-to-use plugin interface that allows arbitrary custom Python scripts to interact with PyKNOSSOS at runtime.

#### PyKNOSSOS: Orthogonal tracing mode and arbitrary reslices

In the default configuration, PyKNOSSOS has five viewports (Fig. 4a). Image data is displayed in four viewports: the YX viewport (imaging plane) and three mutually orthogonal viewports of arbitrary orientation. In tracing mode, one of the latter is perpendicular to the current tracing direction. We find that this ‘auto-orthogonal’ view increases tracing speed and facilitates the identification of branch points and synapses. The reslice views are generated by a tri-linear interpolation procedure to efficiently extract reslices at arbitrary orientations and zoom levels. The fifth viewport displays the skeleton reconstructions.

#### PyKNOSSOS: synapse annotation mode

Synapses can be annotated by three successive clicks to place connected nodes onto the first neuron, the synaptic cleft, and the second neuron. If skeletons of the two neurons are available, the first and the last click connect to existing skeleton nodes. The first click can be omitted if a skeleton node is active. Synapses can be assigned to user-defined classes and scored with a confidence level.

#### PyKNOSSOS: visualization

All visualizations are done using the VTK-Python bindings. Skeleton nodes can be rendered either as points or spheres and skeleton edges can be rendered as lines or tubes. For simple volume representation of convex objects such as somata or olfactory glomeruli, convex hulls can be rendered from sets of nodes.

### Neuron reconstruction

#### Manual tracing

Skeletons of neurons were traced manually using KNOSSOS or PyKNOSSOS. Tracings of OB cells were initiated from seed points close to somata. When cells showed obvious features of glia such as sheet-like processes, tracings were abandoned. A small number of cells was excluded because their morphology was not neuron-like (usually no or only minor processes) or because they did not extend processes into neuropil regions of the OB. All reconstructions of OB cells with neuron-like morphology were completed and included. Starting from seed points, tracers followed neuronal processes by placing successive nodes onto cross-sections of neurites in the original image data, resulting in a skeleton representation of each neuron^27^. Most skeletons were reconstructed by a professional tracing service (www.ariadne-service.ch).

#### Consolidation of skeletons and error correction

Each neuron was initially reconstructed three times by different individuals. Multiple tracings were then combined into a ‘consolidated’ skeleton by CORE (‘COnvergence by Redundancy and Experts’), a procedure based on redundancy (multiple independent tracings of each neuron) and focused input by an expert. During the CORE procedure, errors were identified and corrected. The procedure is described in detail elsewhere^26^. Briefly, individual skeletons were resampled to a node density of approximately 100 nm. Starting from seeds, nodes of skeletons were iteratively combined into ‘cliques’ based on their distance and connectedness. The resulting cliques thus represented the agreement between independent tracings and formed the basis of the consolidated skeletons. Disagreements between one skeleton and the others resulted in nodes that were not part of a clique. To resolve such disagreements, the point of divergence (‘mismatch point’) between the skeletons was inspected by a scientific expert. The expert usually recruited two additional tracers to generate additional tracings of local segments around the mismatch point. In rare cases, particularly when errors were obvious, the expert intervened directly and overruled the tracers. A new consolidated skeleton was then computed and the procedure was repeated until all mismatch points were resolved^26^.

#### Annotation of glomeruli

Glomeruli were defined as distinct neuropil regions containing axons of olfactory sensory neurons, which were identified by their characteristic dark cytoplasmic staining^46^. Seventeen glomeruli were outlined manually in 3D by placing vertices around glomerular boundaries in PyKNOSSOS (Fig. 2e). Because glomerular boundaries were sometimes difficult to delineate when adjacent glomeruli were not separated by somata the outlines may not be fully precise. Sixteen glomerular outlines corresponded in size, shape and position to a previous light microscopic description of glomeruli in the larval zebrafish OB at similar or slightly earlier developmental stages^47,48^. These glomeruli were subdivided into seven groups and named according to the nomenclature of Braubach *et al.*^47,48^ (dG group: dG; dlG group: dlG; vmG/vaG group: vmG, vaG; vpG group: vpG_1_, vpG_2_; lG group: lG_3_, lG_4_, lG_x_; mdG group: mdG_1_, mdG_2_, mdG_3_, mdG_4_, mdG_5_, mdG_6_; maG group: maG). The seventeenth glomerulus was assigned to the mdG group and named mdG_x_ based on its position. In addition, we identified an elongated neuropil volume below lG_3_ and between dlG and vpG_2_ that contained sensory axons. This volume was not included in the set of glomeruli because it was less distinct and smaller than other glomeruli. Furthermore, the central OB contained a neuropil region that was not classified as a glomerulus because we did not detect axons of olfactory sensory neurons. This volume may correspond to the plexiform layer of the adult OB.

### Code availability

The code for PyKNOSSOS and a manual can be found at https://github.com/adwanner/PyKNOSSOS. See Usage Notes for instructions to install and run PyKNOSSOS.

## Data Records

### Dataset 1: EM image stack

Dataset 1 (Data Citation 1) contains 4,746 serial EM images in 8-bit TIF format covering the OB of a zebrafish larva at 4.5 dpf and parts of the adjacent telencephalon (374 GB; Fig. 2a–c). The voxel size is 9.25×9.25×25 nm^3^. Images have been acquired, stitched and aligned as described in Methods.

#### Completeness

The volume contains >99.96% of an entire OB and parts of the adjacent telencephalon. The remaining 0.04% of the OB volume is located in glomerulus mdG_6_ and accounts for <3.97% of the volume of mdG_6_. One section was lost (between slices 1301 and 1302).

#### Data access

The image data is available under http://doi.org/10.7281/T1MS3QN7 and can be accessed through the neurodata web services (NeuroData; http://neurodata.io/wanner16). Data can either be viewed interactively through the NeuroDataViz web interface (http://viz.neurodata.io/WannerAA201605/) or using PyKNOSSOS (Fig. 3). NeuroData provides various APIs to extract and download specific subvolumes (‘cutouts’) for external analysis. A detailed description of the services is available at http://neurodata.io/wanner16.

#### Data viewing in PyKNOSSOS

The repository containing PyKNOSSOS code (https://github.com/adwanner/PyKNOSSOS) includes a dataset configuration file (in folder Datasets/wanner16) to access EM image data hosted by neurodata web services from a local PyKNOSSOS installation. After installation of PyKNOSSOS the dataset can be accessed by loading the configuration file from the menu entry ‘Datasets’ in the ‘Settings’ window. The program automatically fetches the cubed image data around the current location using the online JPEG stack service of NeuroData. By default, PyKNOSSOS uses one download stream but the user can increase the number of parallel download streams in the ‘Loader’ tab of the ‘Settings’ window. PyKNOSSOS uses a hybrid approach for online image data streaming. Any downloaded image cube is stored permanently on the local disk in the same folder as the configuration file (Fig. 3). This minimizes the data streaming traffic because each image cube has only to be downloaded once. In addition, the permanent caching enables offline browsing of already visited locations.

### Dataset 2: Skeleton reconstructions of neurons in the OB

Dataset 2 (Data Citation 2) contains manual skeleton reconstructions and soma outlines of 1,022 neurons in the larval zebrafish OB (101 MB; Fig. 2d). Each reconstructed neuron has a unique ID and is provided in the native PyKNOSSOS file format *.nmx. An NMX-file is a zip-archive container in which annotations and skeletons are saved in individual NML-files, the native KNOSSOS file format, which is XML-based^27^. Each skeleton consists of 3D nodes (vertices) connected by edges. Soma outlines are computed from convex sets of 3D points.

#### Completeness

The 1,022 reconstructed neurons in the dataset represent approximately 98% of all neurons in the OB^26^.

#### Data access

Data can be downloaded from http://dx.doi.org/10.5281/zenodo.58985. Further information including a link to the download site is given on a website hosted by NeuroData (http://neurodata.io/wanner16).

#### Data viewing using PyKNOSSOS

Download and extract the WannerAA201605_SkeletonsGlomeruli.zip from the location specified above. Each NMX-file with the file name pattern Neuron_id*.nmx contains the soma outline and reconstructed skeleton of the neuron with the indicated unique ID. To load one or multiple of these NMX files, start PyKNOSSOS, click on the ‘Open’ command in the ‘File’ menu of the ‘Settings’ window, and select one or multiple files. Multiple skeletons can be loaded simultaneously or sequentially by appending new skeletons to the already loaded ones. Load image data as described above (Dataset 1) to overlay skeletons onto raw data. Various display options for skeletons and somata can be found in the ‘Skeleton’ and ‘Soma’ tab of the ‘Visualization’ tab of the ‘Settings’ window, respectively.

### Dataset 3: Manually outlined volumes of glomeruli in the OB

Dataset 3 (Data Citation 2) contains the outlines of 17 glomeruli that are represented as convex hulls around manually placed 3D points (Fig. 2e). The point sets are provided in the native PyKNOSSOS file format *.nmx. Each glomerulus has an unique ID and a label according to the nomenclature of Braubach *et al.*^47,48^.

#### Completeness

The 17 glomeruli include all glomeruli annotated by Braubach *et al.*^47,48^ at a similar developmental stage and one additional glomerulus (mdG_X_)^26^. In addition, we noticed an elongated neuropil volume below lG_3_ between dlG and vpG_2_ that contained sensory axons. This volume was not included in the set of glomeruli because it was less distinct and smaller than other glomeruli.

#### Data access

Data can be downloaded from http://dx.doi.org/10.5281/zenodo.58985. Further information including a link to the download site is given on a website hosted by NeuroData (http://neurodata.io/wanner16).

#### Data viewing using PyKNOSSOS

Download and extract the WannerAA201605_SkeletonsGlomeruli.zip from the location specified above. The file Glomeruli.nmx contains the outlines of all glomeruli. Use the ‘Open’ command in the ‘File’ menu of the ‘Settings’ window to load the file in PyKNOSSOS. To view glomeruli together with skeletons and image data we recommend to adjust the glomerular region opacity in the ‘Region’ tab of the ‘Visualization’ tab in the ‘Settings’ window.

### Dataset 4: Glomerular innervation pattern of OB neurons

Dataset 4 (Data Citation 2) is a spreadsheet that contains a table in *.csv format that lists the neurite length of each neuron in each glomerulus, generated from the information in Datasets 2 and 3. In addition, the table contains information about the identity (ID) and type of each neuron. The following cell types are distinguished: (1) mitral cells (principal neurons of the OB), (2) class 1 INs (small INs with local processes), (3) class 2 INs (larger INs, often with polarized neurites innervating multiple glomeruli), (4) class 3 INs (usually large INs, often with extensive neurites innervating multiple glomeruli), (5) unusual projection neurons (UPNs; four neurons with IN-like neurites that project out of the OB), (6) large olfactory bulb cells (LOCs; two very large cells with neuron-like morphology but unusual ultrastructure), and (7) other cells that could not be classified unambiguously. IN classes 1–3 were distinguished by clustering of morphological features. For more information see ref. 26.

#### Data access

Data can be downloaded from http://dx.doi.org/10.5281/zenodo.58985. Further information including a link to the download site is given on a website hosted by NeuroData (http://neurodata.io/wanner16).

#### Data viewing using PyKNOSSOS

**Figure 5:**
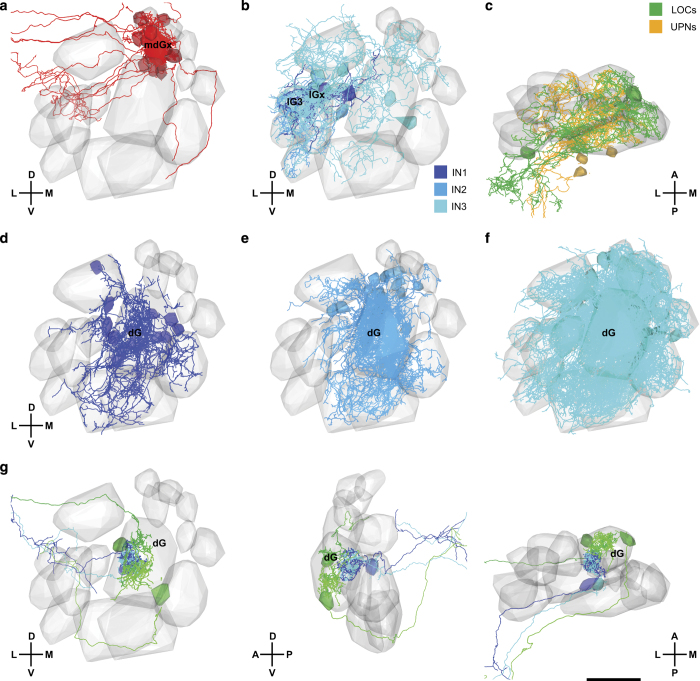
Visualization of neuronal subsets using PyKNOSSOS and the NeuronLibrary plugin. (**a**) Mitral cells with >50 μm neurite length in glomerulus mdG_X_. (**b**) INs of classes 1, 2, and 3 with >50 μm neurite length in lG_3_ and lG_X_. (**c**) Unusual projection neurons (UPNs) and large olfactory bulb cells (LOCs). (**d**) Class 1 INs with >30 μm neurite length in dG. (**e**) Class 2 INs with >100 μm neurite length in dG. (**f**) Class 3 INs with >100 μm neurite length in dG. (**g**) Examples of mitral cells innervating different subcompartments of dG, viewed in three different orientations. Scale bar: 20 μm.

Download and extract the WannerAA201605_SkeletonsGlomeruli.zip from the location specified above. The file WannerAA201605.csv contains a table that lists the neurite length of each neuron in each glomerulus. The csv-file can be loaded into PyKNOSSOS using the NeuronLibrary plugin (Fig. 4b). This plugin facilitates various operations such as filtering of neurons by cell type or glomerulus (Fig. 5).

## Technical Validation

The signal-to-noise ratio and resolution of image data is clearly sufficient to trace neurites and identify synapses. Conductive sample embedding enabled data acquisition in high vacuum. In tests performed with comparable samples, high vacuum conditions increased the signal-to-noise ratio of EM images by a factor of three or more compared to low vacuum conditions (14 or 20 Pa water pressure)^26^, which have been used in most previous SBEM applications^6,18^. No noticeable image distortions were seen throughout most of the dataset because tile sizes were small. Occasionally, small local distortions occurred in regions where the density of conductive material is low. However, these distortions do not affect image interpretation. High quality of image alignment throughout the volume was confirmed by orthogonal reslices and specific inspection of stitching boundaries^26^.

Neuron reconstruction is error-prone and requires error correction procedures to obtain high accuracy. In order to achieve efficient error correction we initially generated three independent reconstructions of each neuron. Errors were then identified and corrected by CORE, a semi-automated method that involves inspection of mismatch points by an expert and local re-annotation around mismatch points^26^. The final reconstruction accuracy was quantified by different measures: (1) Recall, a measure for errors caused by missed processes; (2) precision, a measure for errors caused by wrongly traced processes; and (3) the ‘relative length error’, a measure that takes into account both types of errors^26^. Accuracy was evaluated against a ground truth generated by highly redundant reconstruction and expert error correction, and by comparing independent reconstructions to each other. Error quantification showed that accuracy was high: typically, the total length of missing or incorrectly annotated processes was <5% of the total neurite length^26^. Additional tracing of ‘orphan’ profiles showed that missed processes were usually short terminal branches, <3.5 μm in length, with few synapses^26^.

## Usage Notes

Only a small amount of the information contained in the image data (Dataset 1) and in the skeleton reconstructions (Dataset 2) has been extracted and analyzed so far^26^. These datasets are unique because they represent the near-complete 3D ultrastructure of an entire brain region and a very dense morphological reconstruction of the neurons contained in this brain region. To facilitate further annotations and analyses of these datasets we describe basic procedures to access, visualize and organize these datasets.

### Installation of PyKNOSSOS and data browsing

1. Download and extract the zip-archive of the latest release of PyKNOSSOS from https://github.com/adwanner/PyKNOSSOS.
2. Run PyKNOSSOS
  - from the Python script ‘PyKnosssos.py’ in a Python interpreter: Make sure that you have installed the necessary Python modules listed at the beginning of the script. Some modules such as QxtSpanSlider and images2gif are non-standard and might be missing in some Python distributions.
  - using the stand-alone binary PyKnossos.exe.
3. To load the image data (Dataset 1), click on ‘Load new dataset’ in the ‘Dataset’ menu of the ‘Settings’ window.
4. Browse to the folder ‘Datasets/wanner16’ that was included in the release zip-archive and select the dataset configuration file ‘wanner16.conf’.
5. If you are in ‘online’ mode, i.e. you are connected to the internet and the option ‘Working offline’ in the ‘Dataset’ menu of the ‘Settings window’ is not checked, the hybrid streaming of image dataset cubes should start immediately.
6. Load skeletons and glomerular outlines as described above (Data Records, Datasets 2 and 3). Reconstructions will be shown together with image data.
7. See the included PyKNOSSOS manual for detailed instructions on how to use PyKNOSSOS.

#### Basic navigation:

- Panning: Keep the left mouse button pressed and move the mouse cursor
- Move perpendicular to the currently active viewport: F/D keys or UP/DOWN keys or scroll with the mouse wheel
- Zoom in/out:
  - ○ with mouse: Keep CTRL+right mouse button pressed while moving the mouse cursor up/down
  - ○ keyboard: +/− keys
  - ○ reset zoom to 0 (native resolution) Space-key

### Filtering and visualization of reconstructed neurons

1. Install and run PyKNOSSOS
2. Download and extract the zip-file ‘SkeletonsGlomeruli.zip’ that contains the datasets 2, 3 and 4.
3. Click on ‘Load plugin’ in the ‘Plugins’ menu of the ‘Settings’ window.
4. Browse to the folder ‘plugins’ that was included in the release zip-archive and select the Python script ‘NeuronLibrary.py’.
5. An ‘Open file…’ dialog will be displayed that allows you to load the desired neuron library file.
6. Browse to the folder where SkeletonsGlomeruli.zip was extracted to and select the csv-file ‘WannerAA201605.csv’.
7. A new dockable window containing a table view of all reconstructed neurons will be displayed.
8. Click on the button ‘Data source’ and browse to the folder where SkeletonsGlomeruli.zip was extracted to.

#### Basic usage:

- Double-clicking on any row will load and display the corresponding neuron in PyKNOSSOS.
- By left-clicking on the table header, you can choose different filter options in order to filter the table.
- The different glomeruli have the two filter options ‘>’ and ‘<=’. Depending on the threshold value in the ‘Inn. thres’ spinbox, these filters show those neurons that have more/less neurite length in the corresponding glomerulus.
- Clicking on the ‘Show’ button will load and show all currently filtered/displayed neurons.
- In order to display the glomerular outlines together with the neurons, check ‘Include glomeruli’.
- Right-clicking on any row allows you to change the display color of the corresponding neuron. If multiple rows are selected (partially), the new color will be assigned to all (partially) selected rows.
- Various display options for skeletons, somas and glomerular regions can be found in the ‘Skeleton’, ‘Soma’ and ‘Region’ tabs of the ‘Visualization’ tab of the ‘Settings’ window, respectively.

### Future perspectives

Our datasets can be further mined to address a wide range of questions in cellular, developmental and systems neuroscience. An obvious next step is to annotate the synapses between reconstructed neurons in order to reconstruct the full wiring diagram, an effort that is underway. Exhaustive dense reconstructions of wiring diagrams have so far been achieved only in C. elegans^2,3^ and in representative subvolumes of very few other circuits^5,7–11,14^. The topological analysis of wiring diagrams is particularly important to understand the function of neuronal circuits whose connectivity cannot be approximated based on topological relationships or other means, as in the OB.

Future work may generate dense volumetric reconstructions of neurons in the zebrafish OB by combining automated segmentation with skeleton reconstructions^7,8,36–39^. This approach uses machine learning methods to over-segment EM image data in 3D, resulting in ‘supervoxels’ representing subvolumes of neurons. Skeletons are then used to merge supervoxels from the same neurons. The generation of skeletons is typically a bottleneck in this procedure because it involves substantial manual labor. The skeletons provided along with our EM image data should therefore greatly facilitate volumetric neuron reconstructions.

Previous studies have mapped glomeruli in the developing zebrafish OB, reported expression patterns of various marker genes, determined projection patterns of mitral cells, and characterized odor responses of glomeruli and OB neurons^43,47–56^. Anatomical information about neurons in the zebrafish OB is, however, incomplete. Most anatomical studies focused on mitral cells^43,49,50,57,58^ but only few IN types have been characterized in detail^31,59^. The detailed morphological information contained in our datasets may therefore be exploited to generate a comprehensive atlas of the zebrafish OB that integrates molecular, anatomical and functional information.

The OB of zebrafish and other species contains glomeruli responding to common odorants, as well as subsets of glomeruli that detect specific odorants with a defined biological function such as pheromones^52,53^. Our datasets provide a unique opportunity to explore whether these classes of glomeruli differ in their cellular composition and microcircuit organization, and to study how these glomeruli interact via intra-bulbar projections. Moreover, the high density of our reconstructions allows for a detailed analysis of the sub-glomerular organization of neuronal microcircuits. Preliminary observations indicate that individual mitral cells do not always innervate entire glomeruli but can be restricted to distinct subcompartments (Fig. 5g)^60^. It may thus be interesting to examine whether these subcompartments reflect distinct microcircuits associated with the same glomerulus.

Datasets described in this paper should be particularly valuable to link the morphology of neurons to their ultrastructure. The high resolution of the EM data allows for the quantitative analysis of subcellular components such as mitochondria, endoplasmatic reticulum (ER), primary cilia and synapses. Features of these organelles may then be mapped onto the 3D reconstructions of individual neurons and compared across neurons. A quantitative analysis of synapses, for example, would provide the opportunity to compare the size and other ultrastructural features of synapses within the same neuron, across neurons of the same type, and across neurons of different types. Additional analyses may then ask whether synapses of the same neuron vary systematically depending on the identity of the synaptic partner. Moreover, it is possible to determine whether mitochondria, ER or other organelles are associated preferentially with specific types of synapses. A combined morphological and ultrastructural analysis of neurons has major potential to discover unknown rules governing the organization of neuronal microcircuits^12,13^.

The development of automated procedures for the reconstruction of neurons from volumetric EM data is an active field that requires ground truth datasets. Such datasets are usually obtained by labor-intensive manual reconstruction and often contain only parts of neurons, which complicates the quantification of reconstruction performance at the level of entire neurons. Our skeleton reconstructions may serve as valuable ground truth because they comprise a large number of neurons that were fully reconstructed with high accuracy. In the future, automated reconstruction may also incorporate statistical information about the geometry of neurons. Such information needs to be extracted from large, high-resolution morphological datasets such as our skeleton reconstructions. Our datasets can therefore serve a resource for the future development of automated reconstruction procedures.

## Additional Information

**How to cite this article:** Wanner, A. A. *et al.* 3-dimensional electron microscopic imaging of the zebrafish olfactory bulb and dense reconstruction of neurons. *Sci. Data* 3:160100 doi: 10.1038/sdata.2016.100 (2016).

**Publisher’s note:** Springer Nature remains neutral with regard to jurisdictional claims in published maps and institutional affiliations.

## Supplementary Material



## Figures and Tables

**Table 1:** Reagents and solutions for EM sample preparation.

*Cacodylate buffer*	0.15 M cacodylate (Sigma Aldrich 20838), pH 7.4
*EM Fixative*	2% paraformaldehyde (EMS 15700), 1% glutaraldehyde (EMS 16300), 2 mM CaCl_2_ (Sigma Aldrich 53704) in cacodylate buffer, pH 7.4.
*RedOs solution*	3% K_4_Fe(CN)_6_ (Sigma Aldrich 31254), 0.3 M ice-cold cacodylate buffer with 4 mM CaCl_2_, 4% aqueous OsO_4_.
*TCH solution*	1% thiocarbohydrazide (Sigma Aldrich 88535) in bidistilled H_2_0. Incubate for 1 h at 60 °C, swirl every 10 min, then cool down to room temperature and pass through a 0.22 μm syringe filter (VWR 514-0072).
*Os solution*	4% OsO_4_ (EMS 1/9100) in bdH_2_O.
*UA solution*	1% uranyl acetate in bdH_2_O.
*Walton's lead aspartate*^61^	0.4% aspartic acid (Sigma Aldrich 1043819) in bidistilled H_2_0, add 0.66% lead nitrate (EMS 17900). Incubate at 60 °C for 30 min and adjust to pH 5.5 with 1 M NaOH (Sigma Aldrich 72068; at 60 °C)
*Epon resin*^18^	Mix 9.25 ml glycid ether 100 (Serva 21045), 6.25 ml 2-Dodecenylsuccinic acid anhydride (DDSA, Serva 20755), 5 ml methyl nadic anhydride (MNA), 0.325 ml *N*-benzyldimethylamine (BDMA, Serva 14835) and degas.
*EE*	EE129-4 (Epo-Tek) components A:B ratio 1.25:1.
